# Chicory R2R3-MYB transcription factors CiMYB5 and CiMYB3 regulate fructan 1-exohydrolase expression in response to abiotic stress and hormonal cues

**DOI:** 10.1093/jxb/erx210

**Published:** 2017-07-20

**Authors:** Hongbin Wei, Hongbo Zhao, Tao Su, Anja Bausewein, Steffen Greiner, Karsten Harms, Thomas Rausch

**Affiliations:** 1 Centre for Organismal Studies (COS) Heidelberg, Heidelberg University, Heidelberg, Germany; 2 College of Horticulture, South China Agricultural University, Guangzhou, China; 3 Co-Innovation Center for Sustainable Forestry in Southern China, College of Biology and the Environment, Nanjing Forestry University, Nanjing, China; 4 Südzucker AG, Obrigheim, Germany

**Keywords:** Abiotic stress, fructan 1-exohydrolases, hairy roots, phytohormone, promoter transactivation, R2R3-MYB transcription factor

## Abstract

In the biennial *Cichorium intybus*, inulin-type fructans accumulate in the taproot during the first year. Upon cold or drought exposure, fructans are degraded by fructan exohydrolases, affecting inulin yield and degree of polymerization. While stress-induced expression of *1-FEH* genes has been thoroughly explored, the transcriptional network mediating these responses has remained unknown. In this study, several R2R3-MYB transcriptional regulators were analysed for their possible involvement in *1-FEH* regulation via transient transactivation of *1-FEH* target promoters and for *in vivo* co-expression with target genes under different stress and hormone treatments. CiMYB3 and CiMYB5 selectively enhanced promoter activities of *1-FEH1*, *1-FEH2a*, and *1-FEH2b* genes, without affecting promoter activities of fructosyltransferase genes. Both factors recognized the MYB-core motifs (C/TNGTTA/G) that are abundantly present in *1-FEH* promoters. In chicory hairy root cultures, *CiMYB5* displayed co-expression with its target genes in response to different abiotic stress and phytohormone treatments, whereas correlations with *CiMYB3* expression were less consistent. Oligofructan levels indicated that the metabolic response, while depending on the balance of the relative expression levels of fructan exohydrolases and fructosyltransferases, could be also affected by differential subcellular localization of different FEH isoforms. The results indicate that in chicory hairy root cultures CiMYB5 and CiMYB3 act as positive regulators of the fructan degradation pathway.

## Teaser

Chicory R2R3-MYB transcription factors CiMYB5 and CiMYB3 activate promoters of *1-FEH* genes (*1-FEH1*, *1-FEH2a*, and *1-FEH2b*) and are co-expressed with their target genes under various abiotic stress and phytohormone treatments.

## Introduction

Fructans are a class of sucrose-derived fructosyl-oligosaccharides and are used as major carbohydrate storage compounds in economically important Asteraceae plant species, whereas they are temporarily deposited in the stem and leaf sheath of temperate cereal plants (Hendry, 1993; Van Laere and Van den Ende, 2002; Verspreet *et al.*, 2013). They are classified into inulin, graminan, levan and neoseries according to the linkage type of the glycosidic bond between the fructosyl residues and the position of the glucosyl unit (Ritsema and Smeekens, 2003). Inulin is a linear fructan that contains exclusively (2,1)-linked fructosyl units and a terminal glucose residue (Van Laere and Van den Ende, 2002). Fructans are widely used in the food industry, e.g. as a low calorie sweetener, prebiotic dietary fiber with antioxidant properties, and fat replacer (Lattimer and Haub, 2010). Furthermore, inulin-type fructans have been reported to act as signals in animals, stimulating immune cell activity through Toll like receptor (TLR)-mediated signaling (Peshev and Van den Ende, 2014). As one of the most important sources of inulin, chicory (*Cichorium intybus*) has become a model species for elucidating the regulatory mechanisms of fructan biosynthesis and breakdown. In chicory, fructans are synthesized from sucrose in the vacuole by the consecutive action of sucrose:sucrose 1-fructosyltransferase (1-SST) and fructan:fructan 1-fructosyltransferase (1-FFT). Inulin degradation is predominantly catalysed by fructan 1-exohydrolases (1-FEH), which sequentially remove the terminal fructose units of inulin chains (Van den Ende *et al.*, 2002). Three isoforms have been identified, 1-FEH1, 1-FEH2a and 1-FEH2b (De Roover *et al.*, 1999; Van den Ende *et al.*, 2000, 2001).

Field-grown chicory plants are frequently exposed to variable environmental stresses such as drought and low temperature that adversely affect plant growth and inulin accumulation. Acclimation to these stress conditions requires orchestration of complex metabolic and developmental adaptations, including changes in root-to-shoot ratio and accumulation of osmolytes and cryoprotectants (Hare *et al.*, 1998). Importantly, fructans have been functionally linked to plant adaption to abiotic stresses such as drought and low temperature through maintaining osmotic homeostasis, protecting plasma membrane lipids and scavenging reactive oxygen species (ROS) (Valluru *et al.*, 2008; Livingston *et al.*, 2009; Stoyanova *et al.*, 2011; Peshev *et al.*, 2013; Peukert *et al.*, 2014).

Expression of chicory fructan exohydrolase genes (*1-FEH1*, *1-FEH2a*/*b*) is affected by developmental signals (van Arkel *et al.*, 2012) and various exogenous stimuli, such as cold exposure (Van den Ende and Van Laere, 2002; Michiels *et al.*, 2004; Kusch *et al.*, 2009*a*), defoliation (Van den Ende *et al.*, 2001), and ABA treatment (Bausewein 2014; Wei *et al.*, 2016). Thus, transcript level of *1-FEH2b* was up-regulated following exposure to end-season cold temperatures in the field (via semi-quantitative RT-PCR), showing the same expression pattern as *1-FEH2a*, in spite of sequence variation in their promoter regions (Dauchot *et al.*, 2015). As coding sequences of the paralogs *1-FEH2a* and *1-FEH2b* share high identity (96%), transcripts for *1-FEH2a* and *1-FEH2b* may be co-monitored by quantitative RT-PCR with a single primer pair (van Arkel *et al.*, 2012; this study).

While fructan exohydrolase genes are thought to be transcriptionally regulated (Van den Ende and Van Laere, 2002), little is known about the transcription factor network mediating developmental and stress signaling. In wheat (*Triticum aestivum*), TaMYB13, a R2R3-MYB transcription factor, has been characterized as a transcriptional activator of the fructan synthesis pathway (Xue *et al.*, 2011; Kooiker *et al.*, 2013); however, R2R3-MYB regulators specifically controlling the fructan degradation pathway have not yet been reported, neither for monocot nor for dicot species. Recently, a chicory R2R3-MYB factor was characterized that activated a broad target gene spectrum, including FAZY genes for inulin synthesis and degradation (Wei *et al.*, 2017).

In general, different families of plant transcription factors (TFs) including APETALA2/ethylene response element binding protein (AP2/EREBP), basic-domain leucine zipper factor (bZIP), NAC (*N*AM, *A*TAF1/2, *C*UC2) and MYB (MYeloBlastosis) factors orchestrate stress-induced gene expression (Nakashima *et al.*, 2009, 2014; Saibo *et al.*, 2009). The nomenclature of MYB TFs is based on an MYB DNA-binding domain that contain up to four imperfect repeats (R, 51–53 amino acid residues) in their N-terminus, comprising 1R-MYB, R2R3-MYB, R1R2R3-MYB, and 4R-MYB, respectively (Dubos *et al.*, 2010). In plants, R2R3-MYB proteins represent one of the largest TF families. The Arabidopsis and rice genomes encode 126 and 109 R2R3-MYB proteins, respectively (Dubos *et al.*, 2010). Being restricted to the plant kingdom, R2R3-MYB TFs are involved in the transcriptional control of multiple processes including primary and secondary metabolism, cell fate and identity, plant development, and responses to biotic and abiotic stresses (Stracke *et al.*, 2001; Dubos *et al.*, 2010; Li *et al.*, 2014). R2R3-MYB TFs regulate expression of their target genes through interacting with specific *cis*-regulatory elements, categorized into two main groups, the MYB-core (C/T)NGTT(G/A) and the AC elements (consensus sequences: ACC(A/T)A(A/C)(T/C) and ACC(A/T)(A/C/T)(A/C/T)) (Prouse and Campbell, 2012; Kelemen *et al.*, 2015).

Motivated by the presence of multiple conserved MYB-core cis-elements ((C/T)NGTT(G/A)) in the promoter regions of chicory *1-FEH1* and *1-FEH2a*/*b* genes, an RNAseq database was searched for putative R2R3-MYB transcription factor sequences. Among 34 candidates, two factors, *CiMYB5* and *CiMYB3*, were selected for further study, based on their co-induction with *1-FEH1* and *1-FEH2a*/*b* upon cold-treatment of the chicory hairy root culture (CiHRC). Their specific functionalities in *1-FEH* regulation were subsequently confirmed via a promoter transactivation assay, using the dual luciferase system. Subsequent expression profiling revealed that in response to several stress treatments and phytohormone applications the expression of *CiMYB5* was consistently associated with *1-FEH* transcript levels, whereas for *CiMYB3* this correlation was less stringent. The putative contributions of *CiMYB5* and *CiMYB3* to the orchestration of stress-mediated fructan degradation are discussed.

## Materials and methods

### Chicory hairy root cultures

Chicory hairy root cultures (CiHRC) were previously established and characterized (Kusch *et al.*, 2009*a*). CiHRCs were maintained at 25 °C in the dark on agar plates containing Gamborg B5 medium mixture, 3% sucrose and 0.8% plant agar (pH 5.8). Root material was subcultured monthly to maintain constant growth. For liquid cultures, two different medium compositions were used. Standard medium (SM) consisted of macro- and micronutrients and vitamins as described for Gamborg B5 medium and contained 3% sucrose as carbon source, whereas induction medium had reduced N content, but increased sucrose content (IM_lowN_, 6% sucrose; Kusch *et al.*, 2009*a*). CiHRCs from the plates were incubated in SM medium for 2 weeks as the stock at 25 °C in the dark on an orbital shaker at 100 rpm, and then were separated into numerous 300 l Erlenmeyer flasks containing 200 ml of SM medium.

### Treatment of CiHRC with abiotic stresses and phytohormones

After 3 weeks of growth, CiHRCs were subject to cold treatment (6 °C), PEG 20% (w/v) treatment (polyethylene glycol 8000, Sigma-Aldrich), or 100 mM NaCl treatment. Four phytohormones were added directly to liquid cultures in the following final concentrations: 10 µM abscisic acid (ABA, Sigma-Aldrich), 10 µM *trans*-zeatin riboside (cytokinin, Sigma-Aldrich), 100 µM indole-3-acetic acid (IAA, Sigma-Aldrich), and 100 µM (±)-jasmonic acid (JA, Sigma-Aldrich). ABA was dissolved at a stock concentration of 10 mM in 100 mM NaOH; the remaining chemicals were dissolved in H_2_O. Mock- and phytohormone-treated samples were harvested following time course treatment. CiHRC samples were homogenized to fine powder in an MM400 ball mill (Retsch, GmbH, Germany) and stored at −80 °C until further use.

### Cultivation and treatments of chicory seedlings

*Cichorium intybus* L. var. Zoom seedlings were grown on vermiculite under long day conditions (16 h light/8 h dark) in the greenhouse. Seeds were watered with tap water until germination, and then seedlings were watered every 4 days with nutrient solution (Gamborg B5 medium including vitamins). Six-week-old seedlings were transferred to a cold room (6 °C) and irrigated with ice water. The taproot, petiole and leaf blade samples were collected 24 h after treatment and frozen in liquid nitrogen. Each replicate represents a pool of corresponding tissues from four seedlings.

### Identification of R2R3-MYB transcription factor candidates from a chicory RNAseq database

To generate an RNAseq database, a mixed RNA sample from chicory hairy roots was sequenced on a HiSeq System (Illumina) by the Deep-Sequencing-Core Facility on Heidelberg Campus (http://www.cellnetworks.uni-hd.de/483065/Deep_Sequencing_Core_Facility1). Subsequent transcriptome assembly was done using SOAPdenovo-Trans (http://soap.genomics.org.cn/SOAPdenovo-Trans.html). Thirty-four R2R3-MYB transcription factors were identified from this RNAseq database. Later we obtained the shotgun deep sequencing of chicory genomic DNA database (see below). The partial cDNA of these candidates was confirmed and assembled to full length in the chicory genomic DNA database. Protein sequence alignment was performed using the T-coffee server (http://tcoffee.crg.cat/apps/tcoffee/do:regular). The phylogenetic tree was generated using the Neighbor-Joining method (http://www.phylogeny.fr/).

### Isolation of promoter sequences from the chicory genomic DNA database

To generate a genomic database, genomic DNA from the chicory variety ‘Zoom’ was sequenced on a HiSeq System (Illumina) by the Deep-Sequencing-Core Facility on Heidelberg Campus (http://www.cellnetworks.uni-hd.de/483065/Deep_Sequencing_Core_Facility1). Sequencing revealed 45 GB (approx. 30 times coverage of the genome). Subsequent assembly was done using SOAPdenovo2 (Luo *et al.*, 2012). The generated contig library was used to search for promoter sequences via a BLAST search (Altschul *et al.*, 1990). Promoter sequences of corresponding genes were retrieved from the database. Sequence specific primers were designed to clone the respective promoter from chicory genomic DNA.

### Cloning of transcription factors and target gene promoters using Phusion DNA polymerase

Gene-specifc primers containing gateway overhangs were designed to amplify the entire open reading frame of *CiMYB1*, *3*, *4*, and *5* from the cDNA library (see below) prepared from chicory taproot samples. Promoter sequences of the corresponding target genes were cloned from the genomic DNA. PCR was carried out using 1 μl cDNA (120 ng) as template, 1 μl primers (10 μM), 0.4 μl dNTPs (10 mM), 4 μl 5× buffer, and 0.2 μl Phusion DNA polymerase in a 20 μl reaction. PCR conditions were 98 °C, 30 s, 1 cycle; 98 °C, 15 s; 60 °C, 1 min, and 72 °C, 1 min, 35 cycles; and 72 °C, 5 min, 1 cycle. The list of primer pairs for cloning is shown in Supplementary Table S1 at *JXB* online. The accession numbers for the promoter sequences were *p1-SST* (EU545648.1), *p1-FFT* (EU545647.1), *p1-FEH1* (KY385878), *p1-FEH2a* (AY323935.1), and *p1-FEH2b* (KY385879).

### Promoter transactivation via dual luciferase assay

Construction of the reporter and effector plasmids was performed with Gateway cloning (Thermo Fischer Scientific, St Leon-Rot, Germany). Generally the PCR product was column-purified and firstly cloned into the Gateway entry vector (pDONR201) using Gateway BP Clonase II enzyme mix (Thermo Fisher Scientific), sequenced, and then transferred into the destination vector (pART7 or pLuc) using Gateway LR Clonase II enzyme mix (Thermo Fisher Scientific). The full-length coding sequences of *CiMYB1*, *3*, *4*, and *5* were cloned into the vector pART7, where all transcription factors were under the control of the cauliflower mosaic virus (CaMV) 35S promoter. The nucleotide sequences for the open reading frame of each transcription factor were confirmed by DNA sequencing (Eurofins, Germany). To conduct the transient expression assay, promoter regions of the respective genes of interest were isolated from genomic DNA using Phusion DNA polymerase, and then ligated into the luciferase vector pLuc and confirmed by DNA sequencing. Transient promoter activation assays were carried out in grapevine (*Vitis vinifera*) suspension cells, using the dual luciferase assay protocol as previously described (Czemmel *et al.*, 2009; Höll *et al.*, 2013). The *Renilla* luciferase plasmid pRluc was used as a normalization control in each transfection experiment. All transfection experiments were independently repeated two to three times. Mean values of firefly (*Photinus pyralis*) and *Renilla reniformis* luciferase ratios are reported as relative luciferase activity with error bars indicating standard deviation (SD).

### Yeast-one-hybrid assay

Yeast-one-hybrid analysis was carried out according to the manufacturer’s instructions (Clontech, Takara Bio Europe). For the generation of bait-specific reporter strains, a fragment of 341 bp (−387 to −727 bp upstream of ATG) of the *1-FEH1* promoter, a fragment of 201 bp (−1 to −207 bp upstream of ATG) of the *1-FEH2a* promoter, or a fragment of 1147 bp (−1 to −1147 bp upstream of ATG) of the *1-FEH2a* promoter was cloned into the pAbAi vector with the Gibson Assembly® Cloning Kit (NEB, Ipswich, MA) with primers listed in Table S1. Each of the *p1-FEH*-pAbAi plasmids was integrated via homologous recombination into the genome of the Y1HGold yeast (*Saccharomyces cerevisiae*) strain (Clontech, Takara Bio Europe), which was selected on uracil-deficient synthetic dropout (SD/-Ura) medium. Different concentrations of Aureobasidin A (AbA) antibiotics were used to eliminate the background of bait strains. *CiMYB3*, *CiMYB4*, and *CiMYB5* cDNAs were cloned through Gibson assembly into the GAL4 (the yeast transcription activation protein) activation vector (pGADT7-AD). These prey constructs were then individually transformed into the bait strain cells. The *in vivo* DNA-binding activity was reflected by the growth status of the transformed yeast cells on leucine-deficient synthetic dropout (SD/−Leu) medium supplemented with an appropriate concentration of AbA antibiotics.

### RNA extraction and cDNA synthesis

Total RNA was extracted from around 80 mg of frozen, homogenized tissue with the GeneMATRIX Universal RNA Purification Kit (EURX, Berlin, Germany) according to the manufacturer’s instructions. For cDNA synthesis, 1 µg of total RNA was reverse transcribed in a 20 µl mixture of oligo (dT) primer, RNase inhibitor, and AMV reverse transcriptase (Roboklon) at 42 °C for 20 min, followed by 45 min at 50 °C.

### Quantitative RT-PCR expression analysis

Transcript levels of genes were determined by quantitative PCR with the SYBR Green method on a Rotor-Gene Q (Qiagen). A 15 μl reaction mixture contained the following components: 5 μl cDNA, 1 μl of each primer (5 μM stock), 1.5 μl buffer, 0.3 μl dNTPs (10 mM each), 5.75 μl water, 0.3 μl JumpStart Taq DNA polymerase and 0.15 μl CYBR Green (1:400 dilution of purchased stock solution of ABsolute™ QPCR SYBR Green Fluorescein Mix, ABgene). The thermal cycling conditions used were 95 °C for 6 min followed by 40 cycles of 95 °C for 15 s, 58 °C for 30 s, and 72 °C for 20 s, followed by a melt cycle with 1 °C increments for 5 s each from 56 to 96 °C. The analysis of melting curves, measurement of primer pair efficiencies, and determination of cycle threshold values, including the calculation of the mean normalized expression of the genes, was conducted using the Rotor-Gene Q Series Software Q 2.0.2 (Qiagen) and the Q-Gene software. The mRNA levels of the studied genes were normalized using the comparative *C*_T_ method, using the expression of one or two reference genes (actin and ribosomal protein L19, RPL19) as internal standard (Maroufi *et al.*, 2010; van Arkel *et al.*, 2012). Primer efficiency was considered valid when calculated efficiency was between 90 and 110% with 100% as an optimum.

### Carbohydrate extraction and analysis via HPAEC-PAD

The extraction of total soluble carbohydrates was described in detail by Kusch *et al.* (2009*a*). Recovery rates for inulin and oligofructans ranged from 92 to 97% as determined by adding defined standard compounds before tissue extraction. Aliquots of the final supernatant were dried in a speedvac concentrator (Bachofer, Reutlingen, Germany). Subsequently, samples were dissolved in HPLC water (VWR Prolabo). High-performance anion-exchange chromatography with pulsed amperometric detection (HPAEC-PAD) was performed to determine inulin profiles, and to quantify glucose, fructose, sucrose, 1-kestotriose (GF2), 1,1-kestotetraose (GF3) and 1,1,1-kestopentaose (GF4). Measurements were made with a Dionex ICS-5000 system with Chromeleon 7.2 software (all components from Dionex). For peak identification, glucose (Merck, Darmstadt, Germany), fructose (Applichem), sucrose (Applichem), 1-kestotriose, 1,1-kestotetraose, 1,1,1-kestopentaose (all Wako Chemicals) and RaftilineST (Orafti, Tienen, Belgium) were used as external standards.

### Statistical analysis of data

Bars indicate mean and standard deviation (SD) of three independent experiments. For statistical analysis, the null hypothesis (i.e. no difference between transcript levels of control and treatment) was tested using one-way analysis of variance (ANOVA), followed by a Tukey’s multiple comparison test (SPSS Statistics version 20.0, IBM Corp., Armonk, NY, USA). Where appropriate, asterisks represent significant differences (*P<0.05, **P<0.001) as determined by Student’s *t*-test.

## Results

### Identification of *R2R3-MYB* candidate genes co-induced with fructan exohydrolase genes in response to cold treatment

**Fig. 1.**
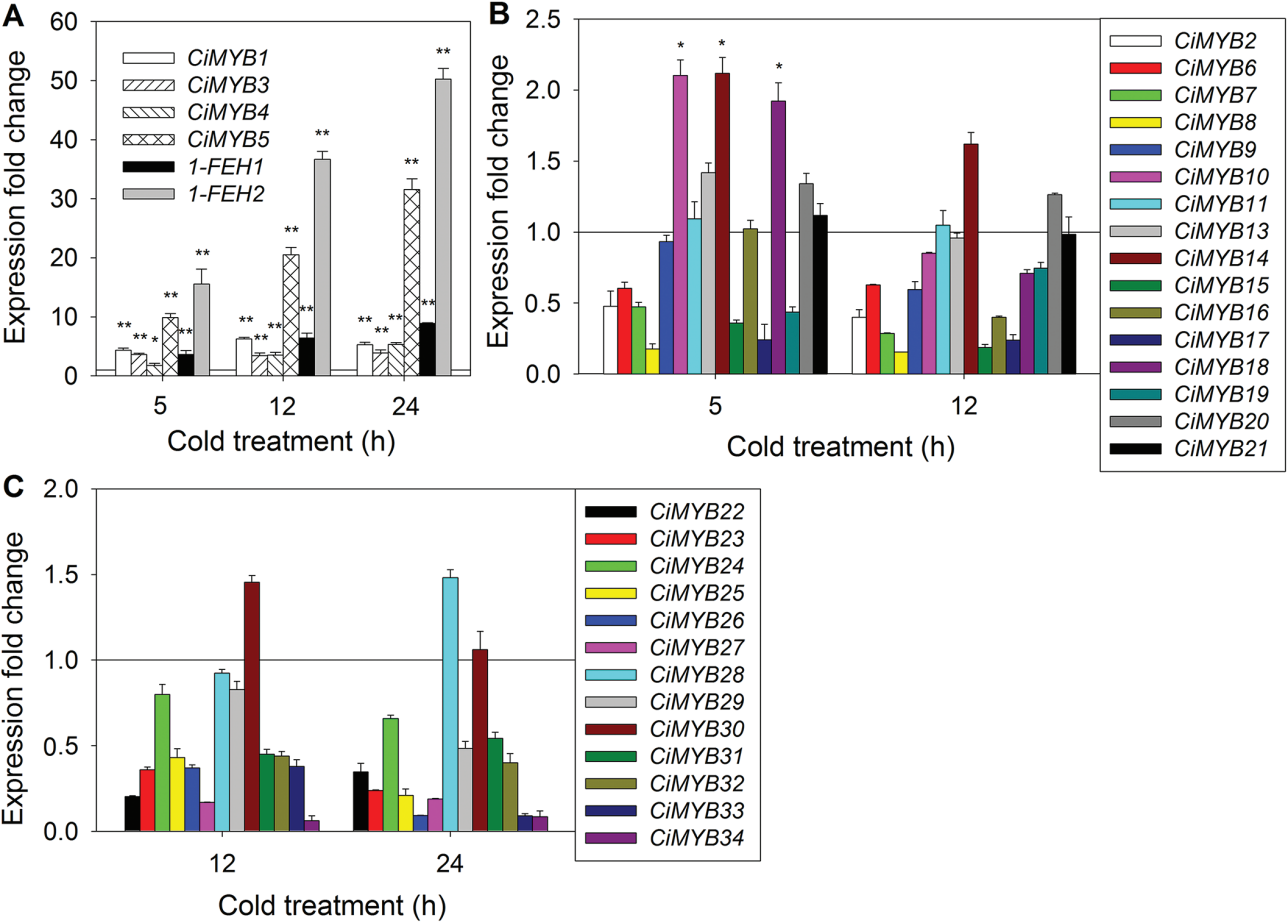
Identification of chicory R2R3-MYB transcription factors co-induced with fructan 1-exohydrolases (*1-FEH1* and *1-FEH2*) by cold treatment. Expression of 34 R2R3-MYB TFs in chicory hairy root cultures (CiHRC) exposed to cold treatment. Transcript levels were detected by qRT-PCR, normalized against expression of RPL19, and expressed relative to those of control samples (0 h), which were set to 1.0 as indicated with a horizontal line. (A) Expression of *CiMYB1*, *CiMYB3*, *CiMYB4*, and *CiMYB5* was strongly co-induced with *1-FEH1* and *1-FEH2*. (B, C) All other R2R3-MYB genes were not co-expressed with *1-FEH* genes. Note that *1-FEH2* transcripts include those of *1-FEH2a* and *1-FEH2b.* Values are means±SD of three independent experiments. Asterisks represent significant difference as determined by Student’s *t*-test (*P<0.05; **P<0.001).

With the goal to identify R2R3-MYB transcription factors regulating the expression of fructan exohydrolase genes, a chicory RNAseq database was generated. Bioinformatic analysis revealed transcripts from 34 *R2R3-MYB* genes. To identify R2R3-MYB candidates for regulation of *1-FEH* genes, expression of all 34 *R2R3-MYB* genes and *1-FEH* genes was monitored by qRT-PCR in chicory hairy root cultures during cold treatment. Among the tested *R2R3-MYB* genes, transcript levels for *CiMYB1*, *CiMYB3*, *CiMYB4*, and *CiMYB5* were strongly up-regulated, being co-induced with *1-FEH1* and *1-FEH2* (10- and 54-fold at 24 h, respectively; note that transcripts for *1-FEH2* include those of both paralogs) (Fig. 1A). Expression of *CiMYB5* displayed the strongest induction (32-fold at 24 h), following a time course similar to that of *1-FEH* genes. Also, induction of *CiMYB4*, while responding less strongly to cold treatment (5-fold at 24 h), revealed a similar time course. Conversely, transcript levels for *CiMYB1* and *CiMYB3* followed a different pattern, i.e. being induced 4- to 5-fold after 5 h cold treatment but thereafter remaining at this level (Fig. 1A). In comparison, expression of *CiMYB10*, *CiMYB14*, and *CiMYB18* was only transiently induced (approximately 2-fold), while expression of the remaining *R2R3-MYB* candidate genes was either unaffected or even down-regulated by cold (Fig. 1B, C). Based on these observations, CiMYB1, CiMYB3, CiMYB4 and CiMYB5 were selected for further analysis.

### CiMYB3 and CiMYB5 activate the promoters of *1-FEH1*, *1-FEH2a* and *1-FEH2b*

**Fig. 2.**
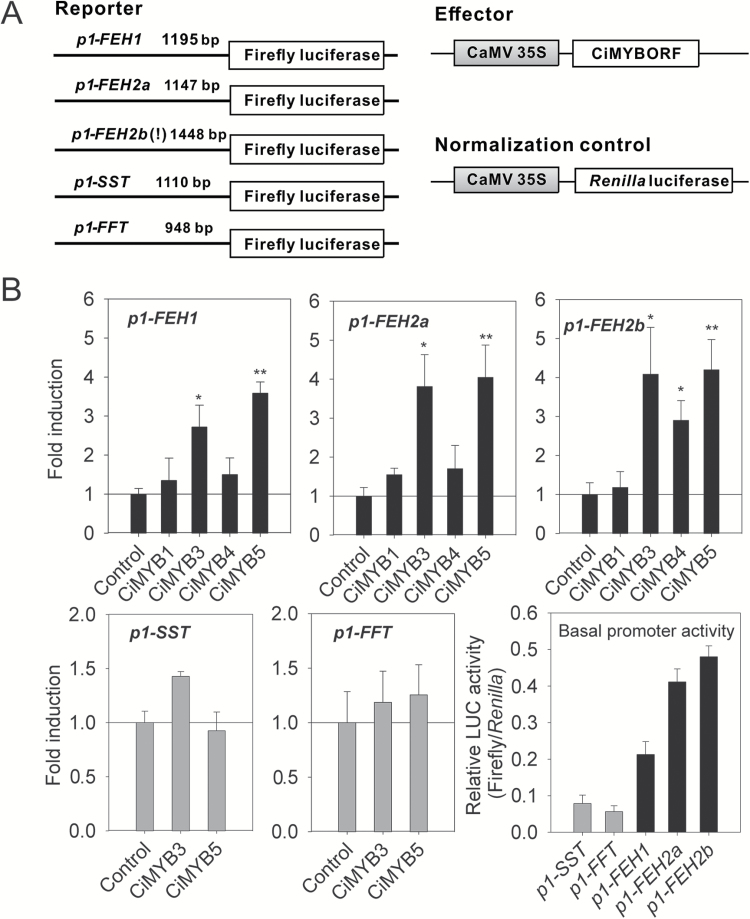
Chicory MYB transcription factors CiMYB3 and CiMYB5 activate the promoters of *1-FEH1* and *1-FEH2a*/*b* but not of fructosyltransferase genes *1-SST* and *1-FFT*. (A) Promoter sequences *p1-FEH1* (1195 bp), *p1-FEH2a* (1147 bp), *p1-FEH2b* (1448 bp), *p1-SST* (1110 bp), and *p1-FFT* (948 bp) were fused upstream of a firefly luciferase gene as reporter construct. The symbol ‘!’ indicates the 1448 bp insertion (−292 to −1740 bp upstream of ATG) of the *p1-FEH2b* as compared with *p1-FEH2a*. Transient transactivation of promoters was performed in grapevine suspension-cultured cells, following particle co-bombardment of the promoter-luciferase construct with the effector construct (pART7-CiMYB; with empty pART7 vector serving as control), and the *Renilla* luciferase plasmid pRluc for normalization of transfection efficiency. Luciferase activity was expressed in arbitrary units relative to the activity of *Renilla* luciferase. (B) Fold induction of FAZY promoter activity in the presence of CiMYB factor, relative to the empty vector control. Basal promoter activities are expressed as relative luciferase activities (firefly/*Renilla*). Bars indicate means±SD of three technical replicates. The results were confirmed in two independent experiments. Asterisks represent significant difference as determined by Student’s *t*-test (*P<0.05, **P<0.001).

**Fig. 3.**
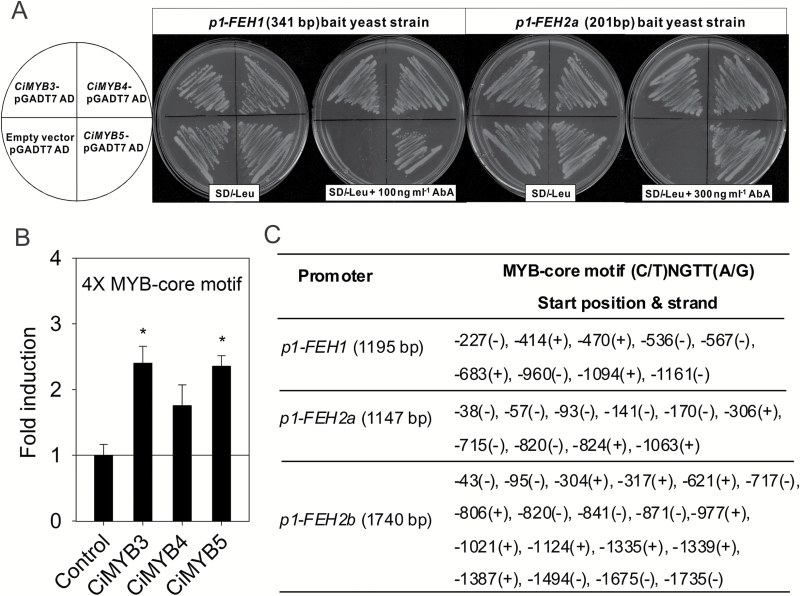
Chicory MYB transcription factors CiMYB3 and CiMYB5 interact with the conserved MYB-core motif (C/T)NGTT(A/G) that is overrepresented in *1-FEH* promoters. (A) For the yeast-one-hybrid assay, a fragment of 341 bp (−387 to −727 bp upstream of ATG) of the *1-FEH1* promoter and a fragment of 201 bp (−1 to −207 bp upstream of ATG) of the *1-FEH2a* promoter were respectively cloned as bait sequences. The concentrations of aureobasidin A (AbA) used to eliminate the background of *p1-FEH1* and *p1-FEH2a* bait strains were 100 and 300 ng ml^−1^, respectively. Yeast cells transformed with CiMYB-pGADT7 plasmid, but not pGADT7 empty vector, were able to grow on leucine-deficient synthetic dropout medium (SD/−Leu) supplemented with AbA antibiotics. (B) One synthetic DNA fragment harboring four copies of the MYB-core motif taken from the *1-FEH1* promoter was sufficient to activate luciferase expression via CiMYB3 and CiMYB5; for further details see Fig. 2. The results were confirmed in two independent experiments. Asterisks represent significant difference as determined by Student’s *t*-test (*P<0.05). (C) Presence of putative MYB-core motif (C/T)NGTT(A/G) in *p1-FEH1* (1195 bp), *p1-FEH2a* (1143 bp), and *p1-FEH2b* (1740 bp). Nucleotide positions are given relative to the translation start codon; the sense and antisense strands are indicated as (+) and (−), respectively.

The competence of CiMYB1, CiMYB3, CiMYB4, and CiMYB5 to activate promoters of *1-FEH1*, *1-FEH2a*, and *1-FEH2b* genes was evaluated via transient transactivation using the dual luciferase assay. Promoter regions of *1-FEH1* (1195 bp), *1-FEH2a* (1147 bp), and *1-FEH2b* (1448 bp) were individually cloned (Fig. 2A). Noteworthy, the promoter region of *1-FEH2b* has a 1.5 kb insertion at position −274 bp as compared with the *1-FEH2a* promoter, while other parts of 1*-FEH2b* and *1-FEH2a* promoter regions share over 88% nucleotide identities. Unfortunately, all attempts to monitor the activities of *1-FEH* promoter-driven reporters in chicory suspension-cultured cells or young seedling leaf blades after particle bombardment were unsuccessful, partially due to very low transformation efficiency. Therefore, transient transactivation assays were performed in a well-established grapevine cell culture system (Höll *et al.*, 2013). As shown in Fig. 2B, CiMYB3 significantly enhanced promoter activities of *1-FEH1* (2.8-fold) and *1-FEH2a* and *1-FEH2b* (3.9-fold). Similarly, CiMYB5 activated promoters of *1-FEH1* (3.7-fold) and *1-FEH2a*/*b* (4-fold), whereas CiMYB1 and CiMYB4 displayed no effect on promoters of *1-FEH1* and *1-FEH2a*; only promoter of *1-FEH2b* was moderately activated by CiMYB4 (Fig. 2B). Therefore, further study focused on CiMYB3 and CiMYB5. Since multiple copies of the MYB-binding core motif are present not only in promoters of *1-FEH* genes but also in promoters of the fructosyltransferase genes *1-SST* and *1-FFT*, possible effects of CiMYB3 and CiMYB5 on the latter promoters were also evaluated (Fig. 2B). Neither transcription factor activated promoters of *1-SST* and *1-FFT*, indicating specificity for promoters of *1-FEH* genes. It is noteworthy that determination of the basal promoter activities (i.e. in the absence of effector constructs) in grapevine cells used for the transactivation assays revealed lower background activities for fructosyltransferase genes as compared with fructan exohydrolase genes, indicating some interaction of endogenous grapevine transcription factors with the latter promoters. To verify that these identified chicory MYB regulators directly bind to *1-FEH* promoters, a yeast-one-hybrid assay was performed. Results showed that both *p1-FEH1* and *p1-FEH2a* bait strains, harboring corresponding partial promoter fragments of 431 and 201 bp, respectively, gained aureobasidin A antibiotic resistance upon expression of *CiMYB3*, *CiMYB4*, and *CiMYB5* (Fig. 3A). It is noteworthy that the bait strain containing 1147 bp of *1-FEH2a* promoter sequence did not effectively detect the binding of chicory MYB transcription factors (data not shown), perhaps due to extremely high yeast background growth, which was completely eliminated when antibiotic concentration reached to 700 ng ml^−1^ (see Supplementary Fig. S1).

### Phylogenetic analysis and molecular features of CiMYB3 and CiMYB5: a comparison with the Arabidopsis R2R3-MYB family

**Fig. 4.**
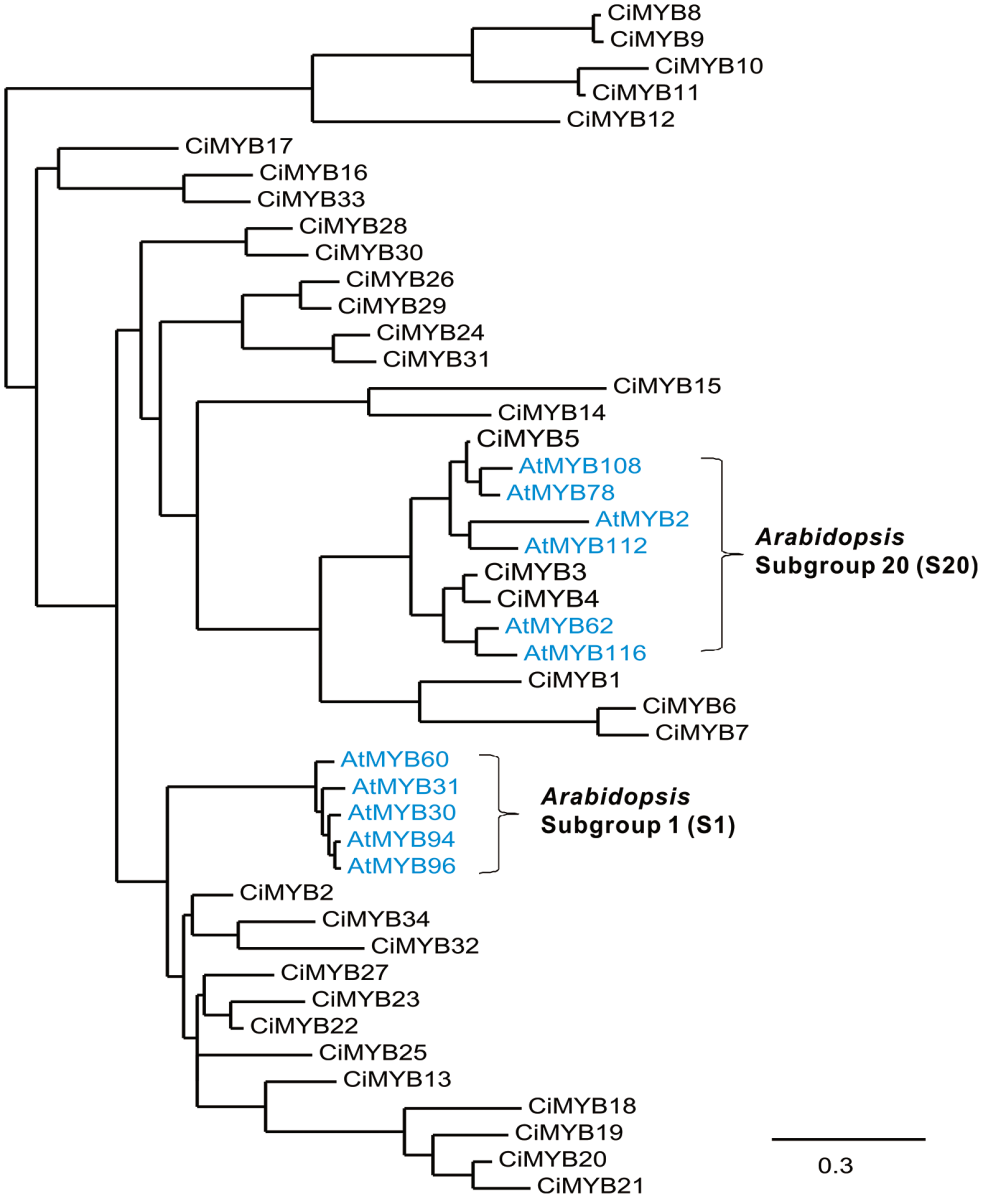
Chicory MYB transcription factors CiMYB3, 4, and 5 are closely related to the Arabidopsis R2R3-MYB subgroup 20 that regulate abiotic stress responses. Phylogenetic relationship of 34 chicory R2R3-MYB factors to Arabidopsis R2R3-MYB factors (in blue color) belonging to subgroup 20 (S20) and subgroup 1 (S1) that are involved in abiotic stress and biotic stress regulation, respectively (Dubos *et al.*, 2010). Phylogenetic analysis was performed with Phylogeny.fr (Dereeper *et al.*, 2010). Accession numbers of chicory R2R3-MYBs: CiMYB1 (KY354365), CiMYB3 (KY354366), CiMYB4 (KY354367), and CiMYB5 (KY354368); Arabidopsis S20 members: AtMYB2 (AT2G47190), AtMYB62 (AT1G68320), AtMYB78 (AT5G49620), AtMYB108 (AT3G06490), AtMYB112 (AT1G48000), and AtMYB116 (AT1G25340); Arabidopsis S1 members: AtMYB30 (AT3G28910), AtMYB31 (AT1G74650), AtMYB60 (AT1G08810.1), AtMYB94 (AT3G47600), and AtMYB96 (AT5G62470.2).

In Arabidopsis the R2R3-MYB family comprises 126 transcription factors, categorized into 25 subgroups based on the conservation of the MYB DNA binding motif and amino acid motifs in the C-terminal domain (Dubos *et al.*, 2010). Proteins in subgroup 20 (S20), including AtMYB2, AtMYB62, AtMYB78, AtMYB108, AtMYB112, and AtMYB116, are reported to regulate abiotic stress responses, while proteins of subgroup 1 (S1), including AtMYB30, AtMYB31, AtMYB60, AtMYB94, and AtMYB96, are mainly involved in biotic stress responses (Kelemen *et al.*, 2015). Phylogenetic analysis revealed that among the identified 34 chicory R2R3-MYB factors, CiMYB3, CiMYB4, and CiMYB5 fall into the same clade with Arabidopsis subgroup 20 (Fig. 4), whereas none of the 34 chicory R2R3-MYB factors displayed sequence similarity with Arabidopsis subgroup 1 proteins. Interestingly, CiMYB3 and CiMYB5 contain a conserved CaM binding motif in their R2 and R3 repeats (Yoo *et al.*, 2005; Supplementary Fig. S2), which is also present in AtMYB2, AtMYB62, and AtMYB78. Further analysis revealed that CiMYB3 and CiMYB5 lack the (D/E)Lx2(R/K)x3Lx6Lx3R signature motif in their R3 repeat, which mediates the protein–protein interaction between R2R3-MYB factors and the N-terminal region of basic helix–loop–helix (bHLH) proteins (Zimmermann *et al.*, 2004; Dubos *et al.*, 2010). Finally, the C-terminal domains of CiMYB3 or CiMYB5 do not contain any ethylene response factor-associated amphiphilic repression (EAR) motif related to those found in R2R3-MYB factors regulating the phenylpropanoid pathway (Cavallini *et al.*, 2015).

Based on the protein sequence similarities of CiMYB3 and CiMYB5 with Arabidopsis subgroup 20, it was of interest to evaluate whether these chicory factors bind to the same *cis*-elements in promoters of their target genes. Since in a yeast-one-hybrid assay members of Arabidopsis subgroup 20 were shown to interact with MYB-core sequences (C/T)NGTT(A/G) (Kelemen *et al.*, 2015), a four-copy repeat of this sequence taken from the *1-FEH1* promoter was synthesized and fused upstream of the coding sequences of firefly luciferase as reporter. As shown in Fig. 3B, both CiMYB3 and CiMYB5 interacted with this DNA fragment, indicating that these chicory factors shared similar DNA binding affinity with Arabidopsis subgroup 20 proteins. Sequence analysis of the promoter regions of *1-FEH1*, *1-FEH2a*, and *1-FEH2b* identified 9, 10, and 19 copies of MYB-core motifs, respectively. Noteworthy, 17 copies are present in the inserted fragment (−292 to −1740 bp upstream of ATG) of *1-FEH2b* promoter (Fig. 3C). These data support the notion that chicory fructan exohydrolases are direct target genes of CiMYB3 and CiMYB5.

### In hairy root cultures, *CiMYB5* is consistently co-expressed with fructan exohydrolases (*1-FEH1* and *1-FEH2*) in response to abiotic stress exposures and phytohormone cues

**Fig. 5.**
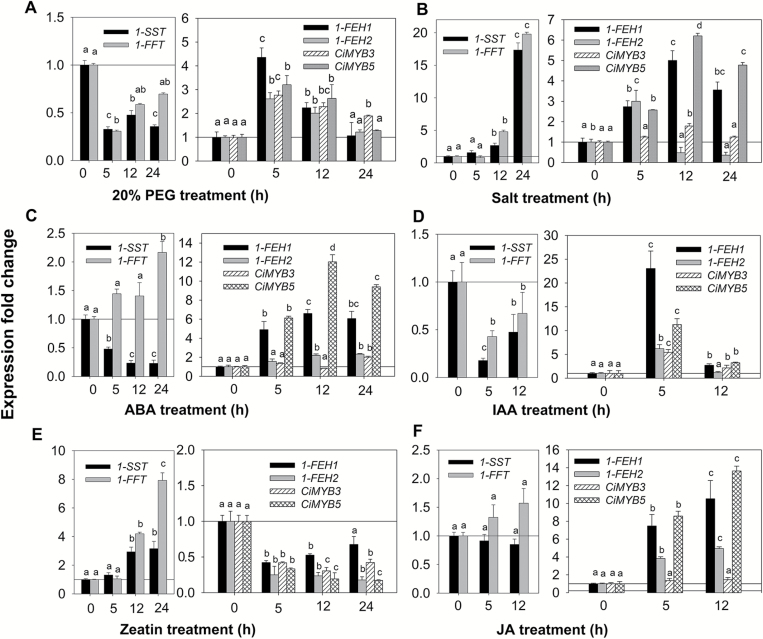
Effect of abiotic stress and phytohormone treatments on expression of FAZYs (*1-SST*, *1-FFT*, *1-FEH1*, and *1-FEH2*) and TFs (*CiMYB3* and *CiMYB5*) in hairy roots cultivated in standard medium. CiHRCs were grown for 14 d in standard medium (SM, 3% sucrose) at 25 °C, followed by different treatment intervals as indicated: (A) osmotic stress induced by 20% polyethylene glycol (PEG); (B) 100 mM NaCl; (C) 10 μM abscisic acid (ABA); (D) 100 μM indole-3-acetic acid (IAA); (E) 10 μM *trans*-zeatin riboside (cytokinin); and (F) 100 μM jasmonic aicd (JA). Transcript levels were detected by qRT-PCR and normalized against two reference genes (RPL19 and actin). Bars indicate means±SD of three independent experiments. Different letters indicate means that differ significantly (*P*<0.05) by one-way ANOVA.

Expanding the initial observation of co-expression under cold exposure (Fig. 1A), effects of additional environmental stimuli (osmotic stress (PEG) and salt stress) and hormonal treatments were explored to further substantiate the functional significance of *CiMYB3* and *CiMYB5* via co-expression with their putative *1-FEH* target genes. Osmotic stress (20% PEG) co-induced the expression of *1-FEH1*, *1-FEH2*, *CiMYB3*, and *CiMYB5*, their transcript levels peaking at 5 h and declining thereafter (Fig. 5A). High salinity (100 mM NaCl) induced *1-FEH1* expression 5-fold, in strong correlation with *CiMYB5* transcript amount (6.2-fold); however, *1-FEH2* transcripts increased only transiently (3-fold at 5 h), followed by down-regulation, whereas *CiMYB3* expression was transiently induced at 12 h (2-fold) (Fig. 5B). It is noteworthy that fructosyltransferase genes (*1-SST* and *1-FFT*) were suppressed by 20% PEG, but were strongly induced by salinity after 24 h.

The role of phytohormones in regulation of chicory FAZY genes is still poorly understood. Since abiotic stress exposure is often intimately connected with hormonal signaling, the impact of several phytohormones (abscisic acid (ABA), indole-3-acetic acid (IAA), cytokinin and jasmonic acid (JA)) on the expression of *CiMYB3* and *CiMYB5* was compared with the response of FAZY genes (Fig. 5C–F). Exposure of hairy roots to 10 μM ABA resulted in a strong increase of transcript levels for *CiMYB5* and *1-FEH1*, peaking at 12 h after onset of treatment (12- and 6.6-fold, respectively), whereas *CiMYB3* and *1-FEH2* were only slightly induced (approximately 2-fold). The two fructosyltransferase genes showed opposite responses upon ABA application, *1-SST* expression being repressed and *1-FFT* expression being up-regulated (Fig. 5C). Treatment of hairy roots with 100 μM IAA resulted in fast but transient increases of transcripts for fructan exohydrolases (23-fold for *1-FEH1*, 6.2-fold for *1-FEH2*) and transcription factors (5.4-fold for *CiMYB3*, 11.3-fold for *CiMYB5*) at 5 h after IAA application, while their transcripts declined at 12 h. In marked contrast, expression of *1-SST* and *1-FFT* was transiently down-regulated (Fig. 5D). In response to cytokinin (*trans*-zeatin) treatment, fructan exohydrolase genes and transcription factors *CiMYB3* and *CiMYB5* were co-suppressed, whereas expression of fructosyltransferase genes was induced (Fig. 5E). Exposure to 100 µM JA induced a strong increase of *1-FEH1* and *1-FEH2* transcript levels (10.5- and 5-fold, respectively, at 12 h), with *CiMYB5* expression displaying a similar induction. Conversely, expression of *CiMYB3* and fructosyltransferase genes was not affected (Fig. 5F). As shown in Fig. S3, the expression of FAZYs (*1-SST*, *1-FFT*, *1-FEH1*, and *1-FEH2*) and transcription factor genes (*CiMYB3* and *CiMYB5*) remained rather stable in control cultures, i.e. in the absence of stress or hormone treatments.

### In hairy root cultures with induced fructan accumulation, co-expression of *CiMYB5* and *FEH* genes is correlated with changes in oligofructan profiles upon stress and hormone treatments

**Fig. 6.**
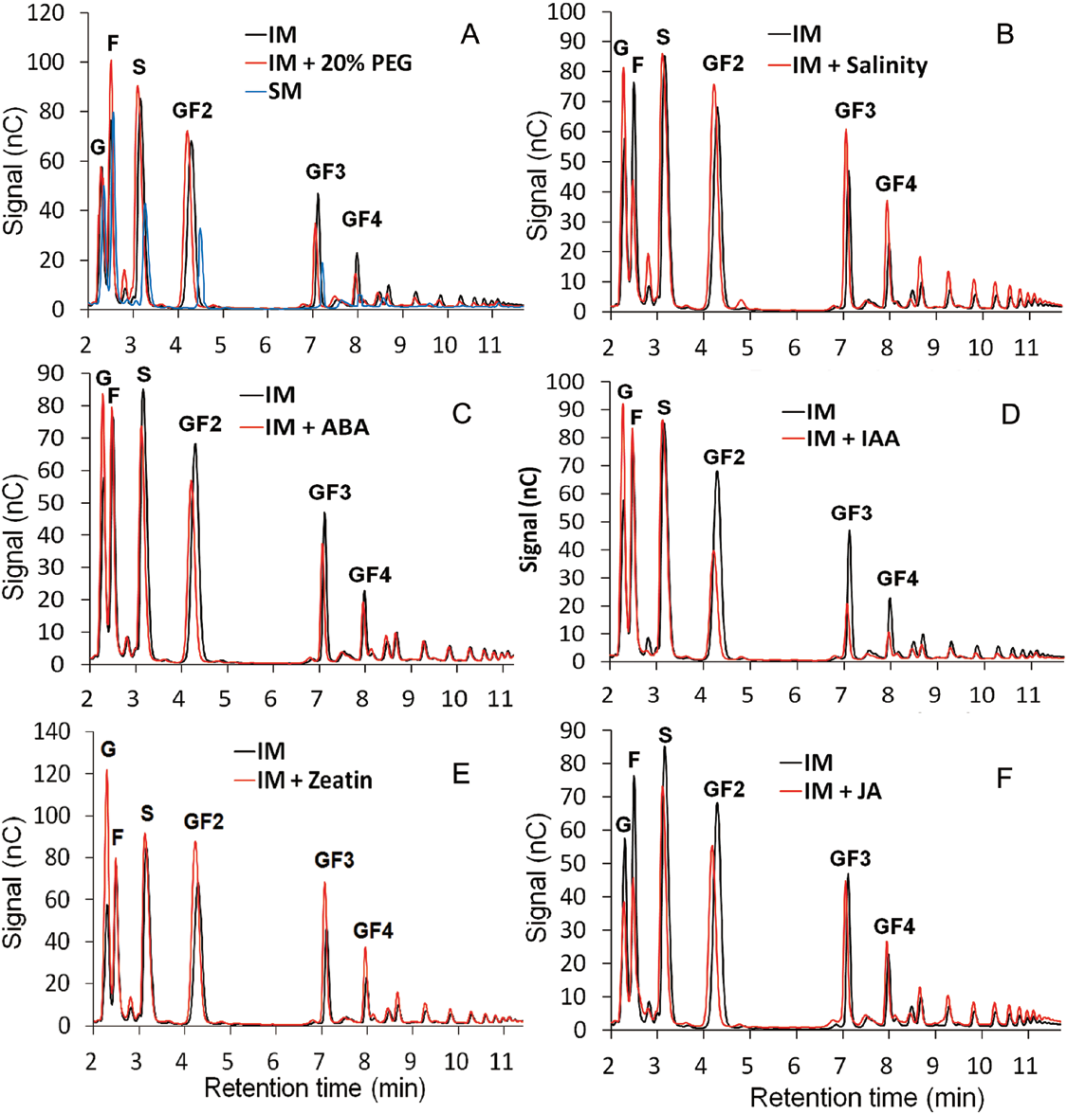
High C/low N-cultivated hairy roots are affected in their oligofructan composition by 72 h abiotic stress exposures or phytohormone treatments. CiHRCs were first grown in standard medium (SM, 3% sucrose) for 1 week and then transferred to inulin induction medium (IM_lowN_, 6% sucrose) for 2 weeks, followed by 72 h of different stress or hormones treatments: (A) osmotic stress induced by 20% polyethylene glycol (PEG); (B) 100 mM NaCl; (C) 10 μM abscisic acid (ABA); (D) 100 μM indole-3-acetic acid (IAA); (E) 10 μM *trans*-zeatin riboside (cytokinin); and (F) 100 μM jasmonic aicd (JA). Carbohydrate measurements were performed via HPAEC-PAD with an ICS-5000 system and Carbpac PA1 column (Dionex). Representative fructan profiles of three independent replicates are shown. F, fructose; G, glucose; GF2, 1-kestotriose; GF3, 1,1-kestotetraose; GF4, 1,1,1-kestopentaose; S, sucrose.

**Fig. 7.**
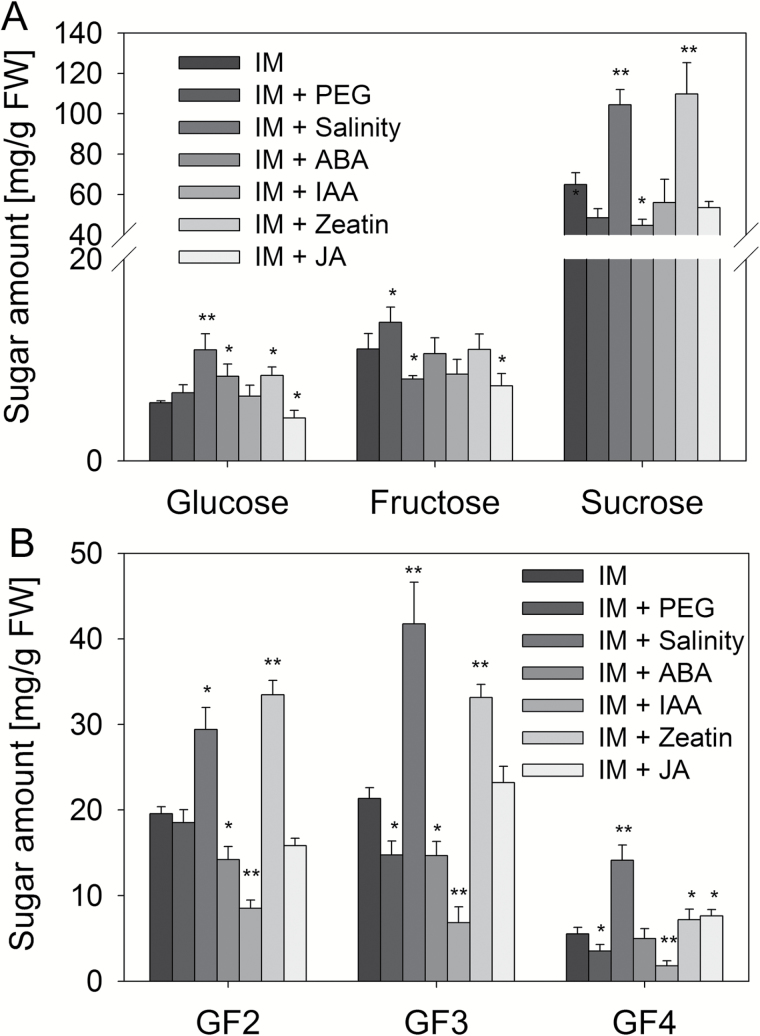
Quantitative analysis of hexoses, sucrose and short chain oligofructans in CiHRCs grown on inulin-induction medium after 72 h exposure to abiotic stress or hormone treatments. Abiotic stress and hormone treatments as in Fig. 6. (A) Contents (mg/g FW) of glucose, fructose and sucrose. (B) Contents (mg/g FW) of 1-kestotriose (GF2), 1,1-kestotetraose (GF3) and 1,1,1-kestopentaose (GF4). Values are means±SD of three independent experiments. Asterisks represent significant differences as determined by Student’s *t*-test (*P<0.05, **P<0.001).

**Fig. 8.**
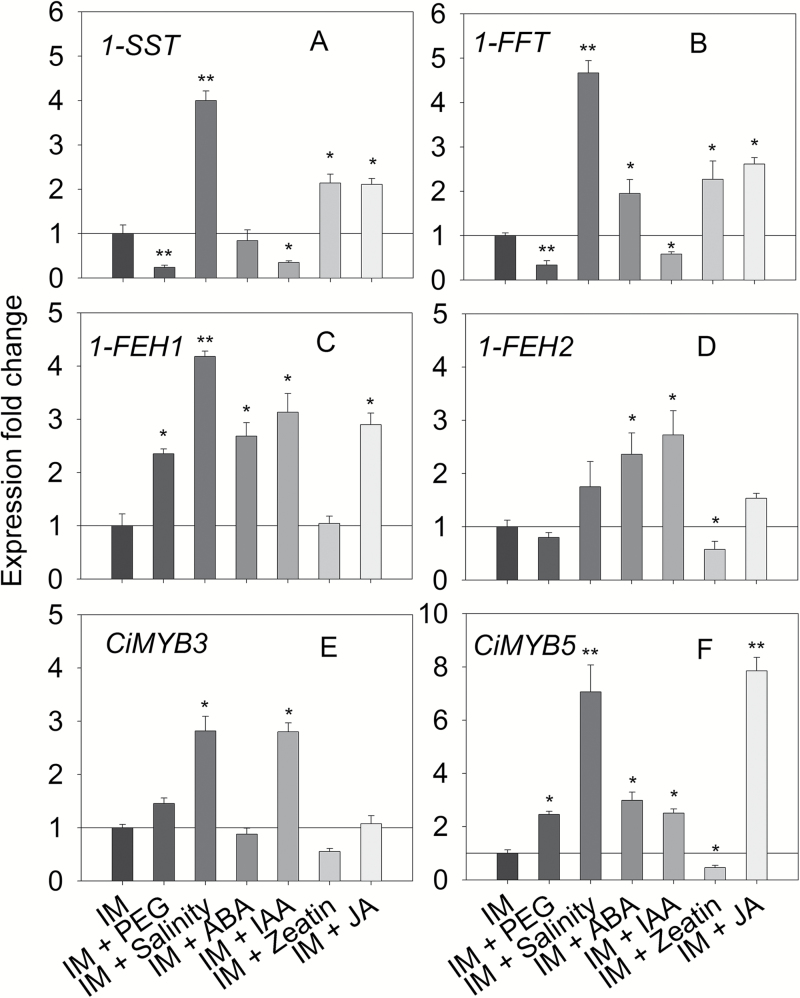
Effects of 72 h exposure to abiotic stress and phytohormone treatments on expression of fructan active enzyme encoding genes (*1-SST*, *1-FFT*, *1-FEH1*, and *1-FEH2*) and chicory MYB transcription factor encoding genes (*CiMYB3* and *CiMYB5*) in hairy roots cultivated in inulin-induction medium. Abiotic stress and hormone treatments as in Fig. 6. Transcript levels were detected by qRT-PCR and normalized against two reference genes (RPL19 and actin). Bars indicate means±SD of three independent experiments. Asterisks represent significant differences as determined by Student’s *t*-test (*P<0.05, **P<0.001).

While the expression profiles of *CiMYBs* under the different stress and hormone treatments (Fig. 5) revealed a rather consistent co-expression of *CiMYB5* with *1-FEH* genes, the correlation was less consistent for *CiMYB3*. Note that for the experiments presented in Fig. 5, chicory hairy roots were cultivated in standard medium (SM, Gamborg B5 medium supplemented with vitamins and 3% sucrose), which did not lead to fructan accumulation (Kusch *et al.*, 2009*a*). In order to explore (i) whether the abiotic stress- and hormone-mediated alterations in FAZY transcripts can accordingly change the fructan profiles, and (ii) whether the observed co-expression of *CiMYB5* with *1-FEH* genes would still be detected under a different nutrition scheme, similar experiments were repeated with hairy roots that had been cultivated in induction medium (IM_lowN_, 6% sucrose) for 2 weeks, causing significant oligofructan accumulation (Fig. 6A). In contrast to the previous set of experiments, stress and hormone treatments were extended to 72 h to allow for metabolic changes in oligofructan levels to be detected (Figs 6–8).

Osmotic stress (20% PEG) resulted in significant oligofructan degradation as shown for GF3 and GF4 (Figs 6A and 7B), the observed changes in oligofructan profiles being correlated with reduced expression of fructosyltransferases (Fig. 8A, B) and increased transcript amounts for *CiMYB5* (Fig. 8F) and *1-FEH1* (Fig. 8C). To the contrary, salt stress (100 mM NaCl) induced inulin biosynthesis (Figs 6B and 7B) due to prominent upregulation of *1-SST* and *1-FFT* expression (Fig. 8A, B) and despite increased expression of *1-FEH1*, *CiMYB3*, and *CiMYB5* (Fig. 8C, E, F). Treatments with ABA (10 μM) and IAA (100 μM) resulted in decreased oligofructan levels (Figs 6C, D and Fig. 7B), correlated with increased expression of *1-FEHs* (Fig. 8C, D) and *CiMYBs* (Fig. 8E, F); note that for IAA treatment, *1-SST* and *1-FFT* transcripts were decreased (Fig. 8A, B), possibly contributing to the lowered oligofructan levels. Cytokinin (10 μM *trans*-zeatin) stimulated oligofructan synthesis (Figs 6E and 7B), correlated with expected induction of fructosyltransferase genes (Fig. 8A, B) and reduced transcript amounts for *1-FEH2*, *CiMYB3*, and *CiMYB5* (Fig. 8D–F). Interestingly, JA (100 μM) substantially increased the amount of high DP (degree of polymerization) fructans, but not of low DP fructans (DP3-4) (Figs 6F and 7B), despite induction of *1-FEH1* (Fig. 8C) and *CiMYB5* transcripts (Fig. 8F); however, transcripts for fructosyltransferase genes (Fig. 8A, B) were also increased. The expression of *CiMYB5* displayed again a better correlation with *1-FEH* expression as compared with its counterpart *CiMYB3*.

### In 6-week-old chicory seedlings cold treatment co-induces the expression of *CiMYB3* and *CiMYB5* with *1-FEH* genes in shoot and taproot

**Fig. 9.**
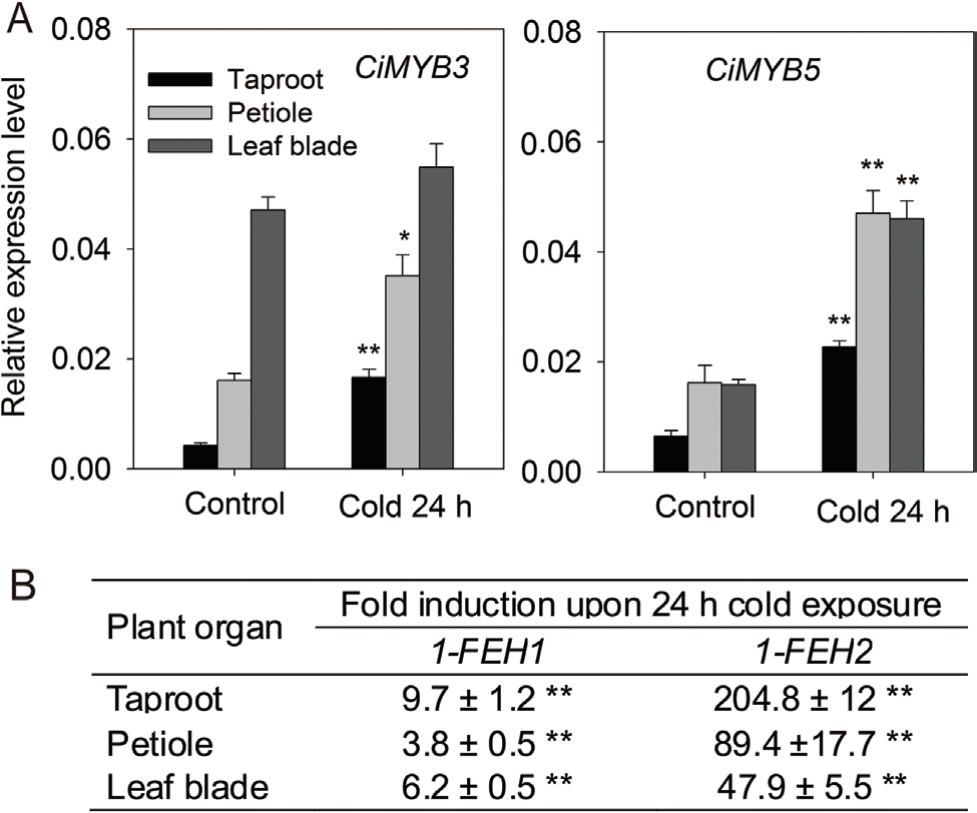
Impact of cold exposure (6 °C) on expression of chicory MYB transcription factor encoding genes (*CiMYB3* and *CiMYB5*) and fructan exohydrolase genes (*1-FEH1* and *1-FEH2*) in different plant organs of 6-week-old chicory seedlings. Six-week-old chicory seedlings were subjected to cold exposure (6 °C) for 24 h. Expression levels were analysed using qRT-PCR and normalized against expression of RPL19. (A) Relative expression levels of *CiMYB3* and *CiMYB5* in different plant organs; note that tissue samples were identical to those analysed by Wei *et al.* (2016). (B) Fold-induction of *1-FEH1* and *1-FEH2* expression in different plant organs in response to cold treatment for 24 h (from Wei *et al.*, 2016). Displayed values are means±SD of three independent experiments. Asterisks represent significant difference as determined by Student’s *t*-test (*P<0.05, **P<0.001).

Cold induction of fructan exohydrolases during chicory cultivation under field conditions has been well established in previous studies (Van den Ende and Van Laere, 2002; Michiels *et al.*, 2004), causing a decline in total inulin yield and degree of polymerization. To examine whether the knowledge gained from the chicory hairy root model system is relevant in the context of real plants, the expression of *CiMYB3* and *CiMYB5* was also determined in 6-week-old chicory seedlings in response to cold treatment (6 °C) for 24 h. Transcripts of *CiMYB3* and *CiMYB5* were moderately induced by cold treatment in taproots and petioles (2- to 4-fold), whereas in leaf blades only *CiMYB5* was induced (2.5-fold; Fig. 9A). In comparison, cold exposure caused a substantial upregulation of transcript levels of *1-FEH1* across different plant organs (ranging from 4- to 10-fold) and *1-FEH2* transcripts (ranging from 48- to 205-fold), being most prominent in the taproot (Fig. 9B). The only moderate induction of *CiMYB3*/*5 versus 1-FEH1*/*2* indicates the involvement of additional regulatory genes.

## Discussion

In chicory, fructan degradation at time of harvest (including pre- and post-harvest degradation) is largely due to the expression of fructan exohydrolase enzymes (1-FEH2a/b, 1-FEH1), in particular 1-FEH2 isoforms, which are induced by cold stress at the end of the growing season. Previous work on chicory (Van Laere and Van den Ende, 2002; Kusch *et al.*, 2009*a*) and durum wheat (Cimini *et al.*, 2015) has shown that fructan active enzymes (FAZYs) display tight correlations between transcript levels and corresponding enzyme activities, indicating that FAZYs are mainly controlled at the transcriptional level. Also, post-translational regulation of FAZYs via inhibitor proteins has been ruled out, as chicory FAZY activities are not inhibited by endogenous chicory invertase inhibitors or invertase inhibitors from other plant species (Kusch *et al.*, 2009*b*), and the existence of invertase inhibitor-related FAZY-specific inhibitor could be ruled out (Bausewein, 2014). Hitherto, studies on chicory and other fructan-accumulating plants have mainly explored how environmental and hormonal cues regulate *FEH* expression (Van den Ende *et al.*, 2001; Michiels *et al.*, 2004; Lothier *et al.*, 2014; Gasperl *et al.*, 2015; Wei *et al.*, 2016), whereas the regulatory genes controlling the fructan degradation pathway have remained largely unexplored. This study has identified two R2R3-MYB transcription factors from chicory that are likely to contribute to expression control of *1-FEH* genes.

### *CiMYB5 and CiMYB3: Cold-induced transcription factors that similarly bind to* 1-FEH *gene promoters but diverge in their response to other abiotic stress cues and hormone treatments*

Based on the results reported in this study, CiMYB5 is proposed to play an important role in the regulation of *1-FEH* genes. This notion is based on the following observations: (1) upon cold treatment, *CiMYB5* expression was co-induced with *1-FEH1* and *1-FEH2* in hairy roots (Fig. 1) and in taproot, petiole and leaf blade of 6-week-old chicory seedlings (Fig. 9); (2) CiMYB5 interacted with promoters of fructan exohydrolase genes in transient transactivation and yeast-one-hybrid assays (Figs 2 and 3A), this effect being rather specific since no activation was observed for promoters of *1-SST* and *1-FFT*; (3) CiMYB5 protein sequence grouped with Arabidopsis subgroup 20 R2R3-MYB transcription factors involved in mediating abiotic stress responses (Fig. 4) (Kelemen *et al.*, 2015); and (4) *CiMYB5* transcripts displayed a consistent co-expression with *1-FEH1* and *1-FEH2*, when hairy roots were exposed to different abiotic stress or hormone treatments, this correlation being observed in two different scenarios, i.e. in the absence (Fig. 5) or in the presence of fructan accumulation (Fig. 8). Conversely, although *CiMYB3* transcripts showed similar co-induction with *FEH* genes in cold-treated hairy roots (Fig. 1) and also similar function in the promoter activation assays (Figs 2 and 3), the correlation with expression of *1-FEH* genes was less consistent during different stress exposures and hormone treatments (Figs 5 and 8). In particular, *CiMYB3* expression was not responsive to abscisic acid or jasmonic acid treatment.

In Arabidopsis, the 126 R2R3-MYB transcription factors are categorized into 25 subgroups based on the conservation of the DNA-binding domain and the variable domain at the C-terminus (Dubos *et al.*, 2010). Arabidopsis TFs belonging to subgroup 20 are well documented to be involved in abiotic stress responses. AtMYB2 regulates responses to ABA, salinity, and drought (Abe *et al.*, 2003) and to phosphate starvation (Baek *et al.*, 2013), whereas AtMYB108 regulates ABA-dependent wound-induced spreading of cell death (Cui *et al.*, 2013). In a previous report it was proposed that binding specificities of R2R3-MYB are related to their biological roles (Kelemen *et al.*, 2015). Therefore, considering the grouping of CiMYB5 and CiMYB3 with Arabidopsis S20 members, the R2R3-MYB factors characterized in this study are proposed to exert similar functions in abiotic stress responses of chicory. It is noteworthy that the calmodulin (CaM)-binding domain is present in the DNA-binding domain of CiMYB3, 4, and 5 and Arabidopsis S20 MYB proteins, indicating post-translational regulation of protein activity in a CaM-dependent manner. Calmodulin, a ubiquitous calcium-binding protein, is one of the best characterized Ca^2+^ sensors. The Ca^2+^–CaM complex is reported to regulate a variety of cellular processes by modulating the activities of numerous target proteins (Kim *et al.*, 2009). A soybean CaM isoform (GmCaM4) was reported to bind to Arabidopsis AtMYB2, in turn enhancing the DNA-binding activity of AtMYB2, and elevating the transcription of AtMYB2-regulated salt and dehydration genes in GmCaM4-overexpression transgenic plants (Yoo *et al.*, 2005). Although CiMYB3 and CiMYB4 are closely related, they demonstrate different activities in transient transactivation assays, which might be partially explained by interactions with the Ca^2+^–CaM complex.

### Induced *1-FEH1* expression coinciding with increased fructan synthesis: an indication of differential compartmentation?

Previous studies have demonstrated that in chicory taproot, fructan synthesis and degradation are temporarily separated following a developmental pattern, impacted by environmental cues such as cold treatment (Van den Ende *et al.*, 2001, 2002). Although enzymes for fructan biosynthesis and degradation were reported to co-localize in the vacuolar compartment (Darwen *et al.*, 1989), later studies revealed that FEHs are evolutionarily related to cell wall invertases rather than to vacuolar invertases (Van den Ende *et al.*, 2002, 2004). Thus, there is as yet no rigorous experimental confirmation that the three characterized 1-FEHs from chicory are vacuolar. It is noteworthy that FEH enzymes, termed defective invertases, are also found in non-fructan plants (De Coninck *et al.*, 2005; Le Roy *et al.*, 2013), suggesting a possible role in the apoplast during plant defense against microbial pathogens. Recently, it has been hypothesized that in fructan accumulating plants, apoplastic fructans released after stress-mediated cell rupture can act as damage-associated molecular patterns (DAMPs) and microbe-associated molecular patterns (MAMPs), contributing to multi-stress resistance potential (Versluys *et al.*, 2017). Also, fructans could be delivered from the vacuole to extracellular space for protecting the plasma membrane by means of vesicle-mediated, tonoplast-derived exocytosis (Valluru *et al.*, 2008).

The assumption of an apoplastic localization of 1-FEH1 would also reconcile the apparent contradiction of its simultaneous induction with fructan biosynthesis enzymes (*1-SST*, *1-FFT*) as observed in salt-treated CiHRCs (Figs 5 and 8). Interestingly, in CiHRCs the expression of *1-FEH1* was strongly increased in response to hormones (ABA and JA), which are also involved in the orchestration of plant defense responses (Fig. 5C, F). However, based on present knowledge vacuolar loacalization of 1-FEH1 cannot be ruled out. In that case, 1-FEH1 might function cooperatively with fructan synthesis enzymes in generating both high DP and lower DP fructans, since a mixture of oligofructans might be optimal for stabilization of the plasma membrane under stress conditions (Valluru and Van den Ende, 2008).

### Promoters of *1-FEH* genes display potential regulatory sequences for interaction with other transcription factor families

Understanding regulatory gene networks controlling various biological processes, including developmental transitions, hormone responses, and abiotic stress responses, requires extensive functional analyses of *cis*-regulatory elements in target gene promoters. Among the best characterized *cis*-elements are the ABA-responsive element (ABRE) and the dehydration-responsive element/C-repeat (DRE/CRT), which function in ABA-dependent and ABA-independent gene expression, respectively, in osmotic and cold stress responses (Yamaguchi-Shinozaki and Shinozaki, 2005; Yoshida *et al.*, 2014). The discrepancy between the only moderate cold induction of the transcriptional regulators CiMYB3 and CiMYB5 and the rather dramatic induction of fructan exohydrolase transcripts in 6-week-old chicory plants (Fig. 9A, B) suggests that additional transcriptional regulators may be involved, which may work synergistically. Sequence analysis of *1-FEH1* and *1-FEH2a*/*b* promoters revealed the presence of additional *cis*-elements, i.e. DRE/CRT (cold and drought responses), ethylene responsive element (ERE), and GCC box (ethylene responses) and W box (abiotic and biotic stress responses), supporting the involvement of additional transcription factors in the regulation of *FEH* genes. As demonstrated in the yeast-one-hybrid assay, bait cells harboring 1147 bp of *1-FEH2a* promoter resulted in higher antibiotic resistance (background growth) as compared with yeast cells harboring shorter (−1 to −201 bp) *1-FEH2a* promoter sequences (Supplementary Fig. S1), indicating that regions between −202 and −1147 bp harbor *cis*-elements that are bound by yeast endogenous proteins, whereas progressive deletion analysis of the *1-FEH2a* promoter indicated that regions from −933 to −717 and from −493 to −278 contain elements that can dampen cold-induced expression (Michiels *et al.*, 2004). Our study also revealed differential regulation of *1-FEH1 versus 1-FEH2* at transcript level, which might be due to the presence or absence of *cis*-regulatory elements in their promoter regions, e.g. the *1-FEH1* promoter lacks the GCC box whereas the DRE/CRT element is absent in the *1-FEH2a* promoter. It is noteworthy that the *1-FEH2a* promoter was predicted to harbor one DREB-like binding site (Michiels *et al.*, 2004); however, transactivation studies revealed that previously identified chicory CiDREB1A and CiDREB1B proteins (Liang *et al.*, 2013) selectively activated *1-FEH1* promoter, but not *1-FEH2a* promoter (Wei and Rausch, unpublished). Furthermore, promoter regions of chicory FAZY genes display the presence of DNA-binding motifs (DTTHGGT, where D=A, G, T and H=A, C, T) for TaMYB13, a transcriptional activator of the fructan synthesis pathway in bread wheat (Xue *et al.*, 2011). Interestingly, another recently identified chicory R2R-MYB factor, CiMYB17, is able to activate both fructosyltransferase (*1-SST*, *1-FFT*) and fructan exohydrolase (*1-FEH1*, *1-FEH2*) genes, via binding to the DTTHGGT *cis*-elements (Wei *et al.*, 2017). In contrast to CiMYB17, CiMYB3, and CiMYB5 selectively activate *1-FEH* promoters despite the presence of MYB-core binding motifs in promoters of fructosyltransferase genes, suggesting that nucleotide sequences flanking the MYB core motif may largely affect the DNA-binding affinity. Together, these R2R3-MYB regulators, and members of other transcription factor families (either transcriptional activators or repressors) are expected to cooperatively or antagonistically regulate the expression of *1-FEH* genes in a stress-dependent manner.

## Conclusions

The results presented in this study, derived from transient promoter transactivation assays, promoter binding assays and extensive expression correlations for transcription factors with their putative target genes, suggest that in chicory hairy root cultures CiMYB3 and CiMYB5 act as transcriptional activators for the expression of *1-FEH1* and *1-FEH2*. The unexpected simultaneous induction of *1-FEH1* with enzymes for fructan biosynthesis as observed in several metabolic scenarios supports the hypothesis that 1-FEH1 may be, in contrast to 1-FEH2, localized in the apoplast; however, this assumption requires experimental verification. Further research is needed to evaluate the contributions of CiMYB3 and/or CiMYB5 in *1-FEH* regulation and 1-FEH-mediated inulin degradation in field-grown chicory taproots at time of harvest, including *in planta* analysis via transgenic approaches.

## Supplementary data

Supplementary data are available at *JXB* online.

Fig. S1. Comparing the background growth (resistance to aureobasidin A (AbA) antibiotics) of different *p1-FEH* bait yeast strains.

Fig. S2. Comparison of the protein sequences of chicory R2R3 MYB transcription factors CiMYB3, CiMYB4, and CiMYB5 with Arabidopsis R2R3 MYB factors belonging to subgroup 20.

Fig. S3. Expression of FAZY genes (*1-SST*, *1-FFT*, *1-FEH1*, and *1-FEH2*) and transcription factor genes (*CiMYB3* and *CiMYB5*) were stable in CiHRCs grown in standard medium (SM, 3% sucrose) during the time-course sampling.

Table S1. Primers used for quantitative real-time PCR analysis, gateway cloning and Gibson assembly cloning.

## Author contributions

HW performed the experiments and wrote the draft. HZ and TS helped with the statistical analysis. AB, SG and KH helped with manuscript preparation and figures. TR supervised the entire study and greatly contributed to the revision of the manuscript.

## Supplementary Material

Supplementary-Figures-S1-S3_Table-S1
