# Supplementary material for: Chicory R2R3-MYB transcription factors CiMYB5 and CiMYB3 regulate fructan 1-exohydrolase expression in response to abiotic stress and hormonal cues
Source: J Exp Bot. 2017 Jul 20;68(15):4323–38. doi: 10.1093/jxb/erx210 (PMC5853547; doi:10.1093/jxb/erx210)
Supplement: Supplementary-Figures-S1-S3_Table-S1 [file erx210_suppl_supplementary-figures-s1-s3_table-s1.pdf]

Supplementary data

Fig. S1 Comparing the background growth (resistance to Aureobasidin A (AbA) antibiotics) of different *p1-FEH* bait yeast strains.

For the generation of bait yeast strains, 341 bp (-387 to -727 bp upstream of ATG) of the *1-FEH1* promoter, 201 bp (-1 to -207 bp upstream of ATG) of the *1-FEH2a* promoter, and 1147 bp (-1 to -1147 bp upstream of ATG) of *1-FEH2a* promoter were respectively cloned into pAbAi vector, which were then integrated into the yeast genome. Positive clones were selected on uracil-deficient synthetic dropout media (SD/-Ura). The concentration of Aureobasidin A (AbA) used to eliminate the background of *p1-FEH1*, *p1-FEH2a_201bp* and *p1-FEH2a_1147bp* bait strains were 100 ng ml^{-1}, 300 ng ml^{-1} and 700 ng ml^{-1} respectively.

Fig. S2 Comparing the protein sequences of chicory R2R3 MYB transcription factors CiMYB3, CiMYB4 and CiMYB5 with *Arabidopsis* R2R3 MYB factors belonging to subgroup 20.

Multiple sequence alignment was performed via *T-coffee* server (http://www.tcoffee.org/). The MYB domain is underlined. The calmodulin binding motif (RWLNYLRPDVRRGNITLE) is indicated in blue color. Asterisks, colons and full stops indicate positions that have an identical, conserved and weakly conserved residue, respectively. Accession numbers refer to Fig. 4.

Fig. S3 Expression of FAZY genes (*1-SST, 1-FFT, 1-FEH1* and *1-FEH2*) and transcription factor genes (*CiMYB3* and *CiMYB5*) were stable in CiHRCs grown in standard medium (SM, 3% sucrose) during the time-course sampling.

Gene expression levels were detected by qRT-PCR and normalized against the expression of two reference genes (RPL19 and Actin). Bars indicate means ± SD of three independent replicates.

Fig. S1

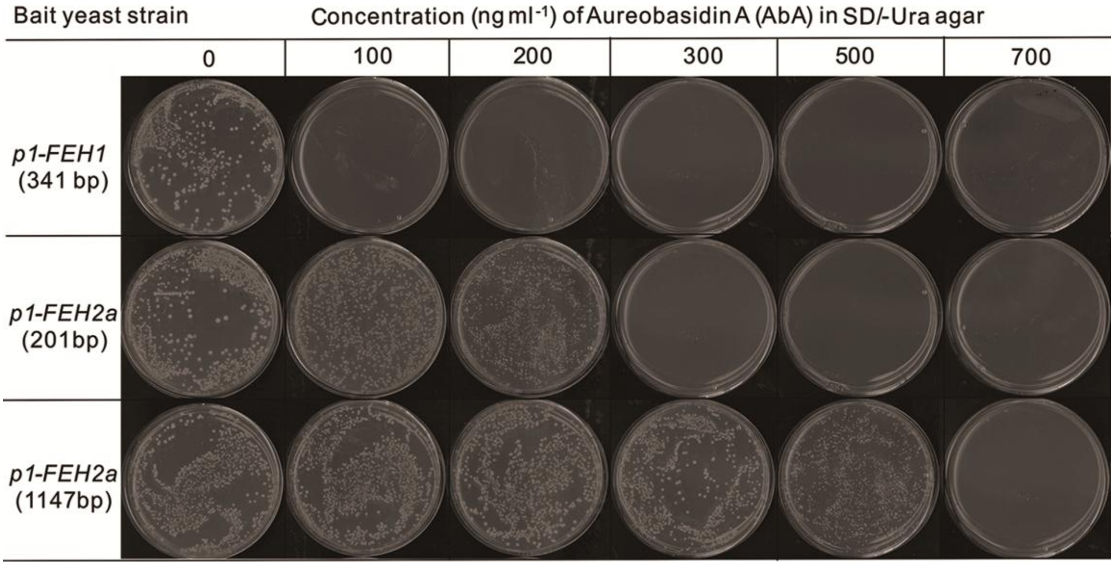
Bait yeast strain
Concentration (ng ml-1) of Aureobasidin A (AbA) in SD/-Ura agar
0
100
200
300
500
700
p1-FEH1
(341 bp)
p1-FEH2a
(201bp)
p1-FEH2a
(1147bp)

Fig. S2

```
BAD AVG GOOD

CiMYB3    MRGTKNMNG-S-------------CNEEEFDQLKRGPWTLEEDNLLIRYITCHGEGRWNSLAKSAGLKRTGK
CiMYB4    MRAAKKTTN-S-------------CNEQEHDELRRGPWTLDEDNLLTHYITCHGEGRWNSLAKSSGLKRTGK
CiMYB5    MNTDNCVDM-GG-----S-EED--DQINMMEDLRRGPWTVEEDFTLINYIAHHGEGRWNSLARCAGLKRTGK
AtMYB62   MENSMKKKK-S-------------FKESEDEELRRGPWTLEEDTLLTNYILHNGEGRWNHVAKCAGLKRTGK
AtMYB116  MSNITKKKC-N-------------G-NEEGAEQRKGPWTLEEDTLLTNYISHNGEGRWNLLAKSSGLKRAGK
AtMYB2    MEDYERINS-NS------------PTHEEDSDVRKGPWTEEEDAILVNFVSIHGDARWNHIARSSGLKRTGK
AtMYB112  MNISRTEFA-NCKTLINHKEEVEEVEKKMEIEIRRGPWTVEEDMKLVSYISLHGEGRWNSLSRSAGLNRTGK
AtMYB108  MDEKGRSLK-------------N--NNMEDEMDLKRGPWTAEEDFKLMNYIATNGEGRWNSLSRCAGLQRTGK
AtMYB78   MGDKGRSLKINK-----NMEEF--TKVEEEMDVRRGPWTVEEDLELINYIASHGEGRWNSLARCAELKRTGK
          *                        .  : ::****  :**   *   ::   :*:.***  :::.:  *:*:**

CiMYB3    SCRLRWLNYLKPDTKRGNLTPQEQLLILELHSKWGNRWSKIAQHLPGRTDNEIKNYWRTRVQKQARHLKIDC
CiMYB4    SCRLRWLNYLKPDVKHGNLTPQEQLLILELHSKWGNRWSKIAQHLPGRTDNEIKNYWRTKVQKQARHLKIDS
CiMYB5    SCRLRWLNYLRPDVRRGNITLEEQLLILELHSRWGNRWSKIAQHLPGRTDNEIKNYWRTRVQKHAKQLKCDV
AtMYB62   SCRLRWLNYLKPDIRRGNLTPQEQLLILELHSKWGNRWSKIAQYLPGRTDNEIKNYWRTRVQKQARQLNIES
AtMYB116  SCRLRWLNYLKPDIKRGNLTPQEQLLILELHSKWGNRWSKISKYLPGRTDNDIKNYWRTRVQKQARQLNIDS
AtMYB2    SCRLRWLNYLRPDVRRGNITLEEQFMILKLHSLWGNRWSKIAQYLPGRTDNEIKNYWRTRVQKQAKHLRCDV
AtMYB112  SCRLRWLNYLRPDIRRGDISLQEQFIILELHSRWGNRWSKIAQHLPGRTDNEIKNYWRTRVQKHAKLLKCDV
AtMYB108  SCRLRWLNYLRPDVRRGNITLEEQLLILELHSRWGNRWSKIAQYLPGRTDNEIKNYWRTRVQKHAKQLKCDV
AtMYB78   SCRLRWLNYLRPDVRRGNITLEEQLLILELHTRWGNRWSKIAQYLPGRTDNEIKNYWRTRVQKHAKQLKCDV
          **********:**  ::*:::  :**::**:**:  ********:::*******:*******:***:*:  *.  :

CiMYB3    SSKRFVEALQRFWMPRLLEKVEQTSSSSSASTA-----STSTMETDQNKLIPYSQSEQTLFAVS-----S--
CiMYB4    NSKRFVEALRQFWPPRLFEKVGHSSPSSFTST--------STMEVDQMK-NPQPQDKETLME----------
CiMYB5    NSKQFKDTMRYLWMPRLVERIQAAATTTATTGGSSSSTTTTTTTSTATTTNSYALNQTNMDCIVTS--QLVV
AtMYB62   NSDKFFDAVRSFWVPRLIEKMEQNSSTTTTYCC-----PQ---NNNNNSLLLPSQSHDSLSMQK-----DID
AtMYB116  NSHKFIEVVRSFWFPRLINEIKDNSYTNNIKAN-----AP--------DLLGP-ILRDSK------------
AtMYB2    NSNLFKETMRNVWMPRLVERINAQSLPTTCEQV-----ESMITDPS------QPVNEP--SPVE---PGFVQ
AtMYB112  NSKQFKDTIKHLWMPRLIERIAATQSVQFTSN----------------------------------------
AtMYB108  NSQQFKDTMKYLWMPRLVERIQSASASSSAAAT-----TTTTTTGSAGTS---------SCITTSNNQFMN
AtMYB78   NSQQFKDTMKYLWMPRLVERIQAASIGSVSMSS------CVTTSS---------------------DQFVI
          .*.  *  :.::  .*  ***.:.:

CiMYB3    PTK--------LINQGSLNS-NSGIRASDSS-GKILPQLPEMPQLY---G--N-----SAFYDSSVQNNDCY
CiMYB4    -----------------NM-NSSSSNSEST-GSKLPH-------------------------AYESSLHCN-SY
CiMYB5    PGG-NNNCGTACSNYGNTQL-NPTYTPENSSTTAVSP-VSDMTDCY------YP---------TNQSQNQEF
AtMYB62   YSG--------FS-----NI-D----GSSST-STCMSHLTTVPHFM---DQSN-----TNIIDGSMCFHEGN
AtMYB116  DLG--------FN-----NM-D----CSTSM-SEDLK---KTSQFMDFSDLET-----TMSLEGSRGGSSQC
AtMYB2    FSQNH------HQQFVP-------ATELSATSSNSP-AETFSDVR------G---------------------
AtMYB112  ---------------------HYSPENSS-VATAT-SSTSSSEA------VR----SS-----------F
AtMYB108  YDYNNNNM---GQQFGVMSN-NDYITPENSS-VAVSP-ASDLTEYY------SAPNPNPEYY-SGQMGNS-Y
AtMYB78   NNNNTNN----VDNLALMSN PNGYITPDNSS-VAVSP-VSDLTECQ------VS----SEVW-K-------I
                                    .  *

CiMYB3    NVDI----------GGFD-MMGLSQ--Q--------DDMSSL-NFQMTD-TDWIS---SG-DMAADTYWNM-
CiMYB4    YVDS----------NNFD-IIGLNQ--PDI-QEFGSGHMSSL-DFQSTD-AEWMN---N---GLVDTFWNI-
CiMYB5    FQQHNTLGIGFT--DAMISPAGYFNQGMDFQ---------AL-VEQ--N-NQWSDGGNGG-DEFSDNLWNV-
AtMYB62   VQEF----------GGY--VPGME-------DYMVNSDISM--ECHVAD-GYSA----YE-DVTQDPMWNV-
AtMYB116  VSEV----------YSS--FPCLEE--EYMVAVMGSSDISALHDCHVAD-SKY-----ED-DVTQDLMWNM-
AtMYB2    --------------------GVVN--GSGY---------DP-SGQ-TG-FGEFND--WG-CVGGDNMWTD-
AtMYB112  YG-GDQ---------------------VEFG---------TL-DHM-TNGGYWFN---GG-D-TFETLCSF-
AtMYB108  YPDQNLVSSQLLPDNYFD-YSGLLD--EDLT---------AM-QEQ-SN-LSWFENINGA-ASSSDSLWNIG
AtMYB78   GQDENLVDPKMTSPNYMDNSSGLLN--GDFT---------KM-QDQ-SD-LNWFENINGMVPNYSDSFWNIG
                                                        .                  :    .

CiMYB3    ---DG--LW----------------  290
CiMYB4    ---DE--LW----------------  265
CiMYB5    ---ED--IWFLKQQ--FN----M---  326
AtMYB62   ---DD--IWQFR-------------E  286
AtMYB116  ---DD--IWQFNEYAHF--------N  283
AtMYB2    --EES--FWFLQDQF-CPDTTSYSYN  273
AtMYB112  ---DELNKWLIQ--------------  243
AtMYB108  ETDEE--FWFLQQQQQFNNNGSF---  323
AtMYB78   -NDED--FWLLQQHQQVHDNGSF---  307
             :   *
```

Fig. S3

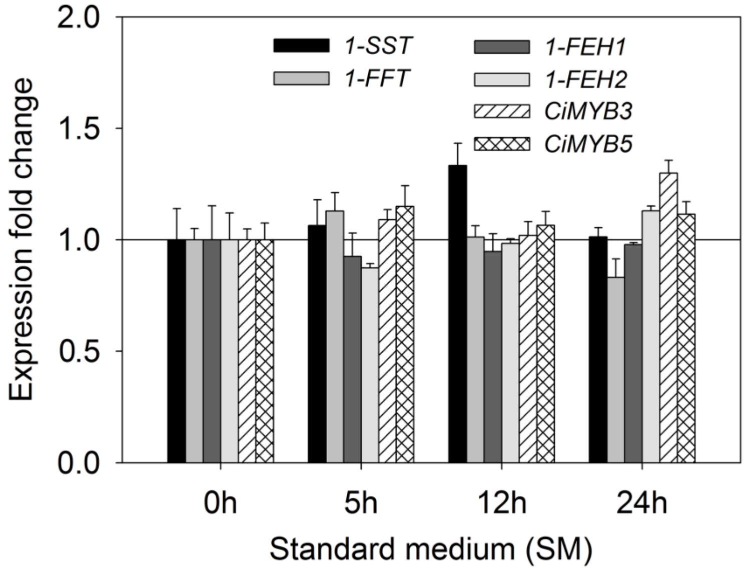

2.0
1.5
1.0
0.5
0.0
Expression fold change
1-SST
1-FFT
1-FEH1
1-FEH2
CiMYB3
CiMYB5
0h
5h
12h
24h
Standard medium (SM)

Table S1. Primers used for quantitative real-time PCR analysis, gateway cloning and Gibson assembly cloning.

The attB1 and attB2 overhangs of gateway primers are underlined. For Gibson assembly, the pGADT7 and pAbAi vectors (Clontech, Takara Bio Company) were linearized by EcoRI and BbsI FastDigest enzymes (Thermo Fisher Scientific), respectively. The overhangs (in small letters) are overlapping DNA fragments with corresponding vector backbone sequences.

**Gene**	**Description**	**Sequence (5‘-3‘)**
**qRT-PCR**		
Actin	F	CCAAATCCAGCTCATCAGTCG
	R	TCTTTCGGCTCCGATGGTGAT
RPL19	F	CTGCCAGCGTCCTCAAGTG
	R	CATTGGGATCAAGCCAAACCT
1-SST	F	CCAACAACCATCAGGGAGGAG
	R	AGCAACGGAGCTGTGAACGT
1-FFT	F	CGGCTACGCAGTTGGACATAG
	R	CTCGTGGTGCAACCGTATTCA
1-FEH1	F	GATAAACGATCCTAACGGAC
	R	GTAGAACAGATGGTAAACTCCA
1-FEH2	F	TAAAGACTTGAAAGAACAAAGTG
	R	TGCACCATAACTTGTCGTGTCG
CiMYB1	F	ACAAGAACAACGACAACAGCAG
	R	CAACCACGATTGGAGGCACATA
CiMYB3	F	GCTGCCTGAAATGCCACAAC
	R	AATCCCATCATATCAAACCCTCCT
CiMYB4	F	GCTGCCTGAAATGCCACAAC
	R	AATCCCATCATATCAAACCCTCCT
CiMYB5	F	GGACACGATGCGGTATCTTTGG
	R	TGGTGGTGGAGGAGGAGGAA

**Gateway cloning**		
p1-SST	GW_attB1	GGGGACAAGTTTGTACAAAAAAGCAGGCTTCTACTATAGGGCACGCGTGGTC
	GW_attB2	GGGGACCACTTTGTACAAGAAAGCTGGGTTGAGTGTGGTAAGGGGTGATTA
p1-FFT	GW_attB1	GGGGACAAGTTTGTACAAAAAAGCAGGCTTCTACGACTTACTATAGGGCACGC
	GW_attB2	GGGGACCACTTTGTACAAGAAAGCTGGGTTTGTTCGAGGGAAATGGGCC
p1-FEH1	GW_attB1	GGGGACAAGTTTGTACAAAAAAGCAGGCTTCCAAAAACCAAAGTTCGTTACCTATTT
	GW_attB2	GGGGACCACTTTGTACAAGAAAGCTGGGTTTCTTTCTGAGCACATTGTTCAGT
p1-FEH2a	GW_attB1	GGGGACAAGTTTGTACAAAAAAGCAGGCTTCCCGCAGACCTCTATCCATATATTAGT
	GW_attB2	GGGGACCACTTTGTACAAGAAAGCTGGGTTCATGATGAGTGTGTGTGTTTGG
p1-FEH2b	GW_attB1	GGGGACAAGTTTGTACAAAAAAGCAGGCTTCGGCTCATAAGTCCTATGGTCGTGAA
	GW_attB2	GGGGACCACTTTGTACAAGAAAGCTGGGTTGAGATTTCTTCATGATGAGTGTGTG
CiMYB1	GW_attB1	GGGGACAAGTTTGTACAAAAAAGCAGGCTTCATGTATTTGGCAGTCATGGGA
	GW_attB2	GGGGACCACTTTGTACAAGAAAGCTGGGTTCTAGCTAGCTTTAGCAGCATGAAAG
CiMYB3	GW_attB1	GGGGACAAGTTTGTACAAAAAAGCAGGCTTCATGAGGGGAACAAAGAACATG
	GW_attB2	GGGGACCACTTTGTACAAGAAAGCTGGGTTTTACCACAATCCGTCCATGTT
CiMYB4	GW_attB1	GGGGACAAGTTTGTACAAAAAAGCAGGCTTCATGAGGGCTGCTAAGAAGACG
	GW_attB2	GGGGACCACTTTGTACAAGAAAGCTGGGTTCCATAATTCATCTATGTTCCAAAAAGT
CiMYB5	GW_attB1	GGGGACAAGTTTGTACAAAAAAGCAGGCTTCATGAATACCGACAACTGTGTGG
	GW_attB2	GGGGACCACTTTGTACAAGAAAGCTGGGTTTCACATGTTGAATTGCTGTTTTAAA
**Gibson assembly cloning**		
CiMYB3	F_pGADT7	tatggccatggaggccagtgCCATGAGGGGAACAAAGAACATG
	R_pGADT7	atcgatgcccacccgggtggTCTTACCACAATCCGTCCATGTT
CiMYB4	F_pGADT7	tatggccatggaggccagtgCCATGAGGGCTGCTAAGAAGACGAC
	R_pGADT7	atcgatgcccacccgggtggTCCTACCATAATTCATCTATGTTCC
CiMYB5	F_pGADT7	tatggccatggaggccagtgCCATGAATACCGACAACTGTGTGG
	R_pGADT7	atcgatgcccacccgggtggTCTCACATGTTGAATTGCTGTTTT
pFEH1 (341 bp)	F_pAbAi	gaattcgagctcggtacATTGGCCTTAATGTCGGACGA
	R_pAbAi	gtcgacagatccccgggtacGACCAGTATACTTGTTTGACCTGT
pFEH2a (201 bp)	F_pAbAi	gaattcgagctcggtacAGTGAGGGGTAGGCCTGGCC
	R_pAbAi	gtcgacagatccccgggtacGATGAGTGTGTGTGTTTGGGG
pFEH2a (1147 bp)	F_pAbAi	gaattcgagctcggtacCCGCAGACCTCTATCCATATATTAGT
	R_pAbAi	gtcgacagatccccgggtacCATGATGAGTGTGTGTGTTTGG
